# The effect of frozen-thawed embryo transfer performed concurrently with hysteroscopy on the reproductive outcomes during assisted reproductive treatments

**DOI:** 10.1038/s41598-017-12068-1

**Published:** 2017-09-19

**Authors:** Xiuxian Zhu, Hongjuan Ye, Yonglun Fu

**Affiliations:** Department of Assisted Reproduction, Shanghai Ninth People’s Hospital, Shanghai Jiaotong University School of Medicine, 639 Zhizaoju Rd., Shanghai, 200000 China

## Abstract

The uterine environment is vital to the successful conception; recently, hysteroscopy was used to remove uterine anomalies in patients undergoing assisted reproductive treatments in combination with a “freeze-all” strategy. However, the rapid recurrence of uterine anomalies impose a negative impact on pregnancy. A possible way to avoid this issue is to implement frozen-thawed embryo transfer (FET) as soon as possible. Thus, we sought to investigate the impact of performing FET concurrently with hysteroscopy in the same mense on the pregnancy outcome. Patients enrolled were divided into two groups: group 1 (n = 272, FET in this mense) and group 2 (n = 251, FET in the next mense). There were no differences in the clinical pregnancy rate (55.15% vs. 53.78%), implantation rate (39.32% vs. 37.2%), spontaneous miscarriage rate (10% vs. 8.89%), or live birth rate (45.96% vs. 45.02%) when comparing the two groups. Binary logistic regression indicated maternal age was negatively associated with the live birth rate, while FET following hysteroscopy in the same mense had no adverse effects on the live birth rate. Our data indicate performing FET concurrently with hysteroscopy in the same menstrual cycle does not impair the pregnancy outcomes, but additional studies with larger populations are needed to confirm these results.

## Introduction

The embryo quality, uterine environment and a combination of both factors play major roles in achieving and continuing a successful pregnancy outcome for patients undergoing assisted reproductive treatments (ART)^1^. Isolated uterine-associated infertility is observed in 2–3% of infertile women, of which intrauterine lesions (40–50%) and endometrial polyps (16–26%) are the most common types of uterine anomalies in these women^2–4^. These uterine pathologies might result in intracavitary bleeding or an abnormal environment for embryo implantation, leading to compromised spontaneous fertility as well as a reduction in pregnancy rates^3^.

Hysteroscopy is generally considered to be the gold standard in uterine cavity assessment, as it makes the direct visualization of the uterine cavity possible. The abnormal adhesions and polyps could be completely cut off without damaging the adjacent endometrium. Hysteroscopy has the advantages of simplicity, low operative, postoperative complications and high patient tolerance^5^. Endometrial pathology was found in 22.9% of 2,500 patients undergoing hysteroscopic evaluation of the uterine cavity before IVF reported in a large prospective study^6^. It was demonstrated that performing hysteroscopy can increase the chance of pregnancy in the subsequent IVF cycle in women who have had one or more failed IVF cycles^7,8^. Hysteroscopic removal of endometrial polyps, submucous fibroids, uterine septum or intrauterine adhesions benefit reproduction and fertility, especially for patients recurrent pregnancy losses^9–11^. Thus, some practitioners recommended a routine hysteroscopy prior to embryo transfer to diagnose and treat such pathologies. Extending the use of hysteroscopy beyond the correction of a uterine pathology was recommended, and this recommendation was corroborated by a recent meta-analysis by Pundir ﻿*et a﻿l.* ^9^, who found a significantly higher clinical pregnancy rate (relative risk (RR) 1.44, 95% CI 1.08–1.92, P = 0.01) and live birth rate (RR 1.30, 95% CI 1.00–1.67, P = 0.05)^9^.

However, it is imperative to highlight that uterine anomalies have a high rate of reformation, which has a significant negative impact on the conception rate^12^. The recurrence rate of uterine adhesions after adhesiolysis was identified to be 3.1%–23.5%, and it increased to 20%–62.5% in severe cases. Additionally, studies demonstrated that the postoperative recurrence rates of endometrial polyps ranged from 2.5% to 43.6%^4^. Factors that account for polyp reformation are still obscure. A high E_2_ level was proposed to be the highest risk factor. Recently, a multivariate logistic regression analysis, including 168 premenopausal women who suffered from endometrial polyps and underwent hysteroscopic polypectomy, revealed that more endometrial polyps (P = 0.015) and a longer duration of follow-up (P = 0.004) were significantly associated with an increased risk of postoperative polyp recurrence^4^.

In prior decades, hysteroscopy was performed prior to controlled ovarian hyperstimulation (COH) when ART relied on fresh embryo transfer (ET). Patients with severe adhesions needed to wait for two menstrual cycles or more because intrauterine device (IUD) was frequently inserted and a second-look hysteroscopy was performed to removed the recurrent adhesions or endometrial polyps. The improvement of embryo vitrification technology and confirmation of its safety made the “freeze-all” policy emerge as an alternative to fresh ET^13,14^. In combination with embryo cryopreservation, embryos could be frozen until the uterine environment was suitable for frozen-thawed embryo transfer (FET) and the endometrium could avoid the super-physiological hormone level due to COH. Therefore, it is necessary to explore the optimal time at which to perform hysteroscopic surgery before embryo transfer. Pereira *et al*. concluded that patients can undergo embryo transfer in the next menses after hysteroscopy without affecting IVF-ET outcomes^15^. In this study, we sought to investigate the impact of performing FET in the same menstrual cycle after hysteroscopy on reproductive outcomes compared with FET in the following menses.

## Results

**Figure 1:**
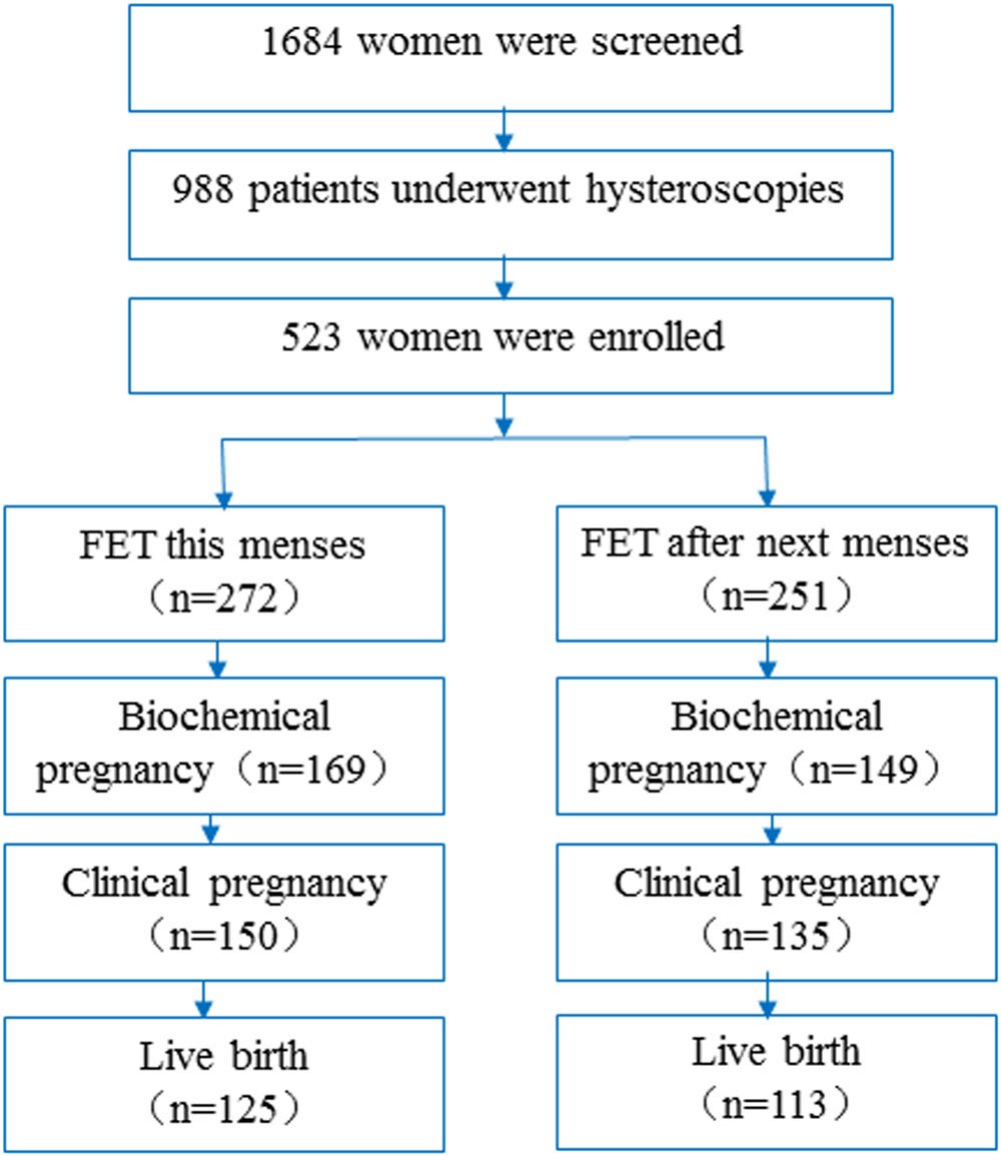
Flowchart of the study.

A total of 1,684 patients underwent FET during the study period. Of the 988 patients who underwent hysteroscopy, 523 patients who met the inclusion criteria were grouped as follows: 272 in group 1 and 251 in group 2. Figure 1 summarizes the selection of the study cohort.

Table 1 compares the demographic and baseline IVF characteristics of patients undergoing FET cycles after hysteroscopy. The mean ages of patients in groups 1 and 2 were 29.22 ± 3 and 29.57 ± 2.85 years of age, respectively. As seen with the mean age, there were no significant differences in the mean gravidity, parity or body mass index (BMI) between the two groups in the study cohort. Furthermore, there were no differences in the basal FSH levels or basal E_2_ levels at the start of FET. Polyps were found in 82 participants in group 1 compared with 67 in group 2, adhesions were confirmed in 145 patients in group 1 compared with 133 patients in group 2, and combined uterine anomalies were observed in 45 patients in group 1 compared with 51 patients in group 2, with no significant difference. Table 2 compares the pregnancy outcomes of patients undergoing FET cycles after hysteroscopy. An embryo transfer was performed approximately 16.5 ± 8.41 days after hysteroscopy in the study group compared to 39.2 ± 4.72 days in the control group. The mean number of embryos transferred were similar among the groups. There was no difference in implantation rates (39.32% vs. 37.2%, p > 0.05), clinical pregnancy rates (55.15% vs. 53.78%, p > 0.05), spontaneous miscarriage rates (10% vs. 8.89%, p > 0.05) or live birth rates (45.96% vs. 45.02%, p > 0.05) when comparing the two groups. One-hundred-eleven patients achieved term delivery in the study group, while 100 patients achieved term delivery in the control group, with no significant difference (p > 0.05). The birth weight and gestation week of the single newborns and twin newborns were comparable between the two groups.

**Table 1:** Baseline characteristics of patients undergoing FET after hysteroscopy.

Parameter	FET in this menses (n = 272)	FET in the next menses (n = 251)	P-value
Age (y)	29.22 ± 3	29.57 ± 2.85	0.21
Duration of infertility (years)	3.26 ± 2.44	3.21 ± 2.34	0.627
Gravidity			0.779
0	176 (64.7%)	169 (67.33%)	
1–2	73 (26.84%)	64 (25.5%)	
≥3	23 (8.46%)	18 (7.17%)	
Parity			0.781
0	250 (91.91%)	229 (91.24%)	
1–2	22 (8.09%)	22 (8.76%)	
BMI (kg/m^2^)	21.87 ± 3.13	21.61 ± 3.4	0.146
Basal FSH (IU/L)	5.34 ± 2.2	5.45 ± 1.78	0.759
Basal E_2_ (pg/mL)	34.95 ± 17.76	33.36 ± 12.39	0.628
IVF indications			0.249
Male factor	29 (10.66%)	23 (9.16%)	
Tubal factor	132 (48.53%)	116 (46.22%)	
Unexplained infertility	9 (3.31%)	3 (1.19%)	
Combination of factors	102 (37.5%)	109 (43.43%)	
Type of uterine anomalies			0.458
Polyps	82 (30.15)	67 (26.69%)	
Adhesions	145 (53.31%)	133 (52.99%)	
Combined anomalies	45 (16.54%)	51 (20.32%)

**Table 2:** Pregnancy outcomes of patients undergoing FET after hysteroscopy.

Parameter	FET in this menses (n = 272)	FET in the next menses (n = 251)	P-value
The interval between hysteroscopy and FET (days)	16.5 ± 8.41	39.2 ± 4.72	<0.001
Thawed embryos (n)	530	493	
Viable embryos after thawed (n)	529	492	
Transferred embryos (n)	1.93 ± 0.26	1.94 ± 0.24	0.962
**The type of transferred embryos**			0.140
No. of cycles with D_3_ embryos (n)	262	247	
No. of cycles with blastocysts (n)	10	4	
**Endometrial preparation (n)**			0.389
Natural cycle	188	172	
Mild stimulation	58	62	
Hormone therapy	26	17	
Endometrial thickness (mm)	11.69 ± 2.85	11.58 ± 2.77	0.408
**Pregnancy outcome of FET (%)**			
Biochemical pregnancy rate per transfer	62.13% (169/272)	59.36% (149/251)	0.517
Clinical pregnancy rate per transfer	55.15% (150/272)	53.78% (135/251)	0.755
Implantation rate	39.32% (208/529)	37.2% (183/492)	0.485
Early miscarriage rate	10% (15/150)	8.89% (12/135)	0.749
Multiple pregnancy rate	36.67% (55/150)	35.56% (48/135)	0.845
Ectopic pregnancy rate	2.67% (4/150)	4.44% (6/135)	0.415
Intrauterine and ectopic pregnancy rate	0.67% (1/150)	0 (0/135)	0.342
**Live-birth outcome**			
Live-birth rate per transfer (%)	45.96% (125/272)	45.02% (113/251)	0.830
Term delivery	111	100	
Preterm delivery	14	13	
Late miscarriage	5	4	
Newborn			
Single newborns (n)	73	67	
Birth weight (g)	3,410.6 ± 491.8	3,237.35 ± 504.63	0.098
Gestational weeks (w)	39.15 ± 1.05	38.7 ± 1.58	0.093
Twin newborns (n)	52	46	
Birth weight (g)	2,544.9 ± 315,31	2,625.21 ± 742.93	0.134
Gestational weeks (w)	36.21 ± 1.29	36.23 ± 2.2	0.193

Table 3 presents the results of binary logistic regression analyses of related factors on the live-birth rate(live birth = 1). The dependent variables comprised maternal age, infertility duration, BMI, whether FET concurrent with hysteroscopy or not(in the same mense = 1, in the next mense = 0), endometrial thickness and number of transferred embryos, which were included in the analysis using the entry method. The final result showed FET following hysteroscopy in the same mense had no adverse impacts on the live birth rate(OR = 1.022, 95% CI: 0.701–1.492). However, a negative effect of maternal age (OR = 0.936, 95% CI: 0.877–1) was identified on the live birth rate.

**Table 3:** Binary logistic regression of factors related to live-birth rate.

Variables	Coefficient(B)	OR (Exp[B]) (95% CI)	Wald (x^2^)	P value
Maternal age (y)	−0.066	0.936 (0.877–1)	3.884	**0.049**
Duration of infertility (y)	−0.003	0.997 (0.919–1.082)	0.004	0.95
BMI (kg/m^2^)	0.011	1.011 (0.955–1.071)	0.14	0.708
Whether FET concurrent with hysteroscopy or not	0.022	1.022 (0.701–1.492)	0.013	0.909
Endometrial thickness (mm)	0.025	1.025 (0.959–1.096)	0.542	0.462
No. of transferred embryos (n)	0.019	1.019 (0.485–2.142)	0.003	0.96

OR, odds ratio; CI, confidence interval; P value shows significance of entrance in the logistic regression model; P values in bold indicate statistical significance.

## Discussion

Uterine anomalies, including endometrial fibroids, polyps, adhesions and uterine congenital abnormalities, can result in abnormal uterine bleeding, amenorrhea or hypomenorrhea, infertility or recurrent pregnancy loss. Hysteroscopy provides an accurate visual assessment of the uterine cavity as well as a chance to treat any pathology detected during the examination. The availability of hysteroscopy with smaller diameters has made the use of outpatient or office hysteroscopy feasible as a routine examination.

Several mechanisms were demonstrated in previous studies with regard to endometrial repair after hysteroscopy. First, an inflammatory response may be conducive to the preparation of the endometrium for implantation. Gnainsky *et al*. collected endometrial samples from women undergoing IVF who had previously failed treatment cycles to determine the abundance of immune cells and the level of cytokines/chemokines^16^. A positive correlation was found between the pregnancy outcome and levels of macrophages/dendritic cells, macrophage inflammatory protein 1B (MIP-1B) expression and tumor necrosis factor-α (TNF-α) expression^16^. These results were corroborated by Junovich *et al*., who observed that the amount of CD56+ uterine natural killer (NK) cells, a main source of endometrial immunoregulatory cytokines, in the endometrium was reduced by ovarian stimulation, but could be normalized by endometrial injury in the late proliferative phase^17^. Second, endometrial gene expression was altered by hysteroscopic therapy. It was confirmed that the mucin-1 transmembrane, laminin a4, matrix metalloproteinase-1, bladder transmembranal uroplakin IB and phospholipase A2 genes, which were responsible for the regulation of cellular proliferation, differentiation and adhesion, were up-regulated^18^. However, An rapid increase in certain factors harmful to the endometrial regeneration and proliferation, such as side population (SP) progenitor cells, were observed by Hyodo *et al*. after endometrial injury in a mouse model^19,20^.

Performing hysteroscopic surgery in patients with intrauterine anomalies diagnosed by HSG or TVS has been accepted as a consensus practice. However, some uterine anomalies are asymptomatic and are prone to be ignored. The prevalence of unsuspected uterine pathology in asymptomatic patients undergoing their first IVF/ICSI treatment varies from 11%–45%^21^. In our study, hysteroscopy was performed prior to embryo transfer in all of the participants undergoing their first embryo transfer. Importantly, scissors, instead of electrosurgical methods, were used to dissect adhesions and excise polyps, as was thermal energy in electrosurgery, leading to tissue vaporization, which may impede the repair of the endometrium^22^.

When should the embryo transfer be implemented after hysteroscopic therapy? Some researchers argue that the short interval between hysteroscopy and embryo transfer may have a negative impact because the endometrium is unable to rapidly regenerate and proliferate. As shown in Karimzade’s study, performing endometrial scratching on the day of oocyte retrieval decreased the clinical pregnancy rate^23^. Nevertheless, it is imperative to highlight that the high rate of recurrent adhesions should be taken into account. Yu *et al*. analyzed the outcome of hysteroscopic adhesiolysis in 85 women with Asherman’s syndrome who presented with a history of infertility or recurrent pregnancy loss. The overall rate of recurrent adhesion was 27.9% at second-look hysteroscopy compared with 41.9% (13 out of 31) in severe cases^12^. In contrast with only two conceptions in 17 severe cases, 26 conceptions occurred in women with normal cavities after a second-look hysteroscopy. The conception rate was affected by the reformation of adhesions after hysteroscopic adhesiolysis^12^.

Thus, several researchers maintain that embryo transfer should be performed as soon as possible. Rana *et al*. divided patients attending an IVF clinic with normal hysteroscopic findings into three groups: hysteroscopy performed 50 days or less before embryo transfer, hysteroscopy between 51 days to 6 months and hysteroscopy at more than 6 months before embryo transfer. The highest pregnancy rate indicated that the endometrium effect was highest when hysteroscopy was performed 50 days or less before embryo transfer^1^. Pereira *et al*. suggested performing embryo transfer in the next menses after hysteroscopy^15^. On the other hand, a retrospective study by Eryilmaz *et al*. concluded that pregnancy outcomes were unrelated to the time interval, as no significant difference was found in the pregnancy rate between hysteroscopy and the initiation of IVF in 29 and 31 patients who underwent IVF <6 months and >6 months after hysteroscopic polypectomy, respectively^24^. Broadly speaking, the optimum time to perform hysteroscopic surgery before embryo transfer is far from resolved.

Our study showed that the clinical pregnancy rate, implantation rate and early miscarriage rate were comparable between the two groups. Moreover, the live-birth outcomes, such as the live-birth rate, birth weight and gestational weeks, showed no differences. This finding illustrates that performing FET in the same menstrual cycle after hysteroscopy will not compromise the reproductive outcomes. Embryo transfers were performed approximately 16.5 ± 8.41 days after hysteroscopy in the study group compared with 39.2 ± 4.72 days in the control group. No association was observed between the time intervals and live birth rate in the binary logistic regression analysis of related factors on live-birth rates. The result of our study, along with the studies by Guven *et al*., maintains that the shortest time between hysteroscopy prior to embryo transfer that is associated with a positive effect is 2 to 3 weeks^25^. This result was corroborated histologically by Li *et al*.’s experiment in animal models, which demonstrated that complete endometrial repair requires approximately two weeks^26^. Furthermore, increased E_2_ levels, as in the case of ovarian stimulation, play a significant role in the development of some uterine anomalies, such as endometrial polyps, which contain functional endometrium associated with estrogenic stimulation^27^. The freeze-all strategy makes FET uniquely geared to cope with the beneficial aspects of hysteroscopic surgery, as surgery performed after the ovarian stimulation was in the absence of a high estrogenic environment. It has been reported that the pregnancy rates and fetal growth-related perinatal outcomes of FET tend to be better than those of fresh embryo transfers^13,14^. Therefore, it is feasible to implement FET after hysteroscopy in the same menstrual cycle.

There are several benefits of performing concurrent FET and hysteroscopy. First, the reformation of adhesions can be minimized such that the best effect of hysteroscopic therapy can be achieved. Second, the period of the treatment can be shortened, as waiting for one or more menstrual cycles after hysteroscopy does not necessarily yield superior outcomes. These findings are especially significant for patients with a poor ovarian reserve, as the time saved means more chances for success. Third, the patient’s anxiety can be relieved without a delay in the completion of treatment, which makes the procedure more simple and efficient.

A major limitation of our study was that it was not a randomized trial, and although the participants were strictly screened, the control cycles were retrospectively matched, which may have limited statistical validity. In addition, the severity of intrauterine anomalies and the time of therapy were not included in the analysis, which may influence the reproductive outcomes.

In conclusion, this study showed that the performance of FET was feasible with hysteroscopy in the same menstrual cycle without impairing pregnancy outcomes. This approach may be especially beneficial for patients who urgently require embryo transfer due to physical or psychological reasons and those who endured reformation of adhesions or polyps in a short time. Further blinded randomized controlled trials should be performed to confirm the feasibility of this method, identification of a suitable population, optimal interval and long-term safety of pregnancy and labor.

## Material and Methods

### Study Setting and Patients

A retrospective study was conducted at the Department of Assisted Reproduction at the Ninth People’s Hospital of Shanghai Jiaotong University School of Medicine. Women undergoing FET cycles after hysteroscopy were recruited from June 2014 to July 2015. The study was conducted according to the Declaration of Helsinki for medical research. Owing to the retrospective nature of the study, individual consent was unnecessary.

The following inclusion criteria were used: 35 years of age or younger; high-quality embryos that were subjected to cryopreservation by vitrification and were still in good condition after being thawed; patients undergoing embryo transfer for the first time; uterine adhesion or polyp discovered by hysteroscopy. All of the hysteroscopies were performed in the follicular phase of the menstrual cycle. Patients received an IUD if a uterine adhesion was found, which often takes two menstrual cycles. The IUD was removed until the uterine environment was found to be normal by the second-look hysteroscopy. Once intrauterine synechia was observed during the second-look hysteroscopy, a repeat adhesiolysis procedure was implemented. Hysteroscopic polypectomy was performed in patients with endometrial polyps. Exclusion criteria included patients with recurrent IVF failure; documented ovarian failure, including a basal FSH level above 10 IU/L or no antral follicles according to ultrasound examination; endometriosis grade 3 or higher; presence of hydrosalpinges; or any contraindications to assisted reproductive treatment.

The study group consisted of 272 patients who completed FET in the same menstrual cycle as the hysteroscopy. The control group included 251 patients who finished FET in the next menses after hysteroscopy.

### Clinical and Laboratory Protocols

Patients underwent COH using the progesterone protocol as described previously^28^. Briefly, human menopausal gonadotropin (hMG) (Maanshan Pharmaceutical Trading Co., Anhui, China) 150 to 225 IU were administered, concurrently, Utrogestan (Laboratories Besins International, Paris, France) 100 mg twice a day were taken orally from menstrual cycle (MC) day 3–5 until the trigger day. Six days later, a transvaginal ultrasound examination and hormone determination were conducted to monitor the development of follicles. The final stage of oocyte maturation was triggered when there were more than 3 dominant follicles reached 18 mm in diameter, using 0.1 mg triptorelin (Decapeptyl, Ferring Pharmaceuticals, Germany) or 3000 IU human chorionic gonadotrophin (hCG) (Lizhu Pharmaceutical Trading Co., Zhuhai, China). Transvaginal ultrasound–guided oocyte retrieval was carried out 34–36 hours after the trigger. All follicles which was more than 10 mm in diameter were picked up.

Based on the specific parameters of semen, *in vitro* fertilization (IVF) or intracytoplasmic sperm injection (ICSI) was performed to fertilize the aspirated oocytes. On the third day after oocyte retrieval, embryo quality was evaluated according to the number and regularity of blastomeres and the degree of embryonic fragmentation, as stated by Cummins *et al*.^29^, while good-quality embryos defined as embryos with more than 6-cell as well as grade 1 or grade 2, were frozen by vitrification. Only non-top-quality embryos were placed in extended culture until they reached the blastocyst stage. During this stage, only good-morphology blastocysts were frozen on day 5 or day 6.

### Endometrium Preparation and FET

For hysteroscopic examination and endometrial mechanical stimulation, the methods and diagnostic criteria followed those published in “Gynecologic Endoscopy” and “Practical Hysteroscopy”^30^.

In this study, embryo and endometrium synchronization with FET was performed via the same methods in the two groups. In brief, natural FET cycles were used for women with regular menstrual cycles, and letrozole was added for patients with irregular menstrual cycles. For patients with thin endometria during either natural cycles or stimulation cycles, hormone treatment was recommended for endometrial preparation. Specifically, red Fematon oral tablets (2 tablets two times per day, including 2 mg micronized estradiol per tablet; Abbott Healthcare Products B.V.) were administered from cycle day 3 onward. After the endometrial lining of each patient was confirmed to be thicker than 8 mm, yellow Fematon tablets were administered orally (2 tablets two times per day, including 2 mg micronized estradiol and 10 mg dydrogesterone per tablet; Abbott Healthcare Products B.V.), and soft vaginal P capsules (200 mg twice per day; Laboratoires Besins International) were also administered. Fematon was designed for sequential therapy for menopausal women, and we used the yellow tablet to initiate secretory changes. The transfer of Day 3 embryos was arranged to be performed 3 days later. The transfer of blastocysts was performed on the fifth day. Once pregnancy was achieved, the exogenous estrogen and P supplements were continued until 10 weeks of gestation.

### Outcome Measures

The primary outcome measure was live birth rate. The delivery of a viable infant was considered as the live birth. A biochemical pregnancy was defined as a positive hCG level without a gestational sac. The presence of a gestational sac with or without fetal heart activity, ectopic pregnancy and heterotopic pregnancy were regard as clinical pregnancy, examined at least 7 weeks after FET. The implantation rate was referred to the number of gestational sacs divided by the number of embryos transferred. The miscarriage rate was reckoned as the proportion of patients with a spontaneous termination of pregnancy.

### Statistical Analyses

In this study, the data were presented as the means ± SDs, number or percentage. The normality of continuous variables was tested using the Shapiro-Wilk test. The Student’s t-test was applied if the continuous variables were normally distributed; otherwise, theMann-Whitney U-test was used for the variables of non-normal distribution, and the chi-square test was used for categorical variables. Binary logistic regression was performed to quantify the effect of related factors on the live birth rate. In the regression analysis, the possible factors in the model included maternal age, infertility duration, BMI, FET following hysteroscopy in the same mense or the next mense, endometrial thickness and the number of transferred embryos. The enter method was employed when these factors were introduced into the regression equation. An adjusted odds ratio (OR) and 95% confidence interval (CI) were adopted to reflect the effect of related factors on the live birth rate. A P-value of <0.05 was considered statistically significant. All data were analyzed using the Statistical Package for the Social Sciences for Windows (SPSS, ver. 16.0).
